# Technical considerations in interpreting rapid transfusion through a 4.2-Fr Broviac catheter

**DOI:** 10.1186/s40981-026-00878-0

**Published:** 2026-10-07

**Authors:** Keiichiro Mizuno

**Affiliations:** Center for Perioperative Services and Critical Care, Fukuoka Children’s Hospital, Fukuoka, Japan

To the Editor,

I read with great interest the valuable case report by Fujioka et al. describing the successful management of acute hemorrhage during nephrectomy for Wilms tumor with inferior vena cava extension [1]. The authors’ multidisciplinary preparation, immediate availability of a rapid infusion device, and transesophageal echocardiographic assessment provide useful practical information for anesthesiologists managing sudden major bleeding in small children.

To facilitate appropriate interpretation of this experience, several technical details would merit clarification. The authors report that a single-lumen 4.2-Fr Broviac catheter was connected to an SL One rapid infusion device and that the infusion rate was intermittently increased to 150 mL/min. It is unclear whether this represented a programmed or measured flow rate. The trimmed catheter length, maximum observed in-line pressure, pressure-limit setting, and occurrence of pressure-related alarms or flow limitation were also not reported.

These details are important because the mechanical conditions of infusion through a small-bore catheter cannot be characterized by the programmed flow rate alone. In an in vitro study using a roller pump, Maki et al. demonstrated that in-line pressure increased with both flow rate and catheter length in a 4-Fr pressure-resistant peripherally inserted central catheter [2]. Although their experimental catheter and infused fluid differed substantially from the Broviac catheter and blood–albumin mixture used in the present case, their findings illustrate the importance of reporting catheter length and circuit pressure when describing high-flow infusion through a small-bore catheter.

The authors state that the SL One monitors infusion pressure, and this function may have been an important safeguard. Clarification of the pressure-control conditions would help readers interpret the mechanical safety of this device–catheter combination. It would also be informative to know whether catheter integrity and function were specifically assessed after rapid infusion, including any leakage, damage, or subsequent dysfunction.

The distinction from manual syringe-based administration is particularly important. Manual syringe-based rapid administration of blood products or fluids through a Broviac catheter may expose the catheter to substantial and poorly controlled in-line pressure and should therefore be approached with particular caution. If resistance does occur, changing to a smaller syringe in an attempt to facilitate injection may generate even higher pressure and further increase the risk of mechanical catheter damage [3]. The reported experience should therefore not be interpreted as supporting such manual syringe-based rapid administration through a Broviac catheter or other methods without comparable pressure-monitoring and pressure-limiting safeguards.

I respectfully suggest that the authors provide the relevant pressure-control and catheter-related details. Such information would help readers better understand the conditions under which this high-flow infusion was achieved and would strengthen the educational value and patient-safety message of this important report.

## Data Availability

Data sharing is not applicable to this article as no datasets were generated or analyzed during the current work.
